# Comparative genomic insights into *Yersinia hibernica* – a commonly misidentified *Yersinia enterocolitica*-like organism

**DOI:** 10.1099/mgen.0.000411

**Published:** 2020-07-23

**Authors:** Scott Van Nguyen, Dechamma Mundanda Muthappa, Athmanya K. Eshwar, James F. Buckley, Brenda P. Murphy, Roger Stephan, Angelika Lehner, Séamus Fanning

**Affiliations:** ^1^​ UCD-Centre for Food Safety, School of Public Health, Physiotherapy & Sports Science, University College Dublin, Belfield, Dublin D04 N2E5, Ireland; ^2^​ Institute for Food Safety and Hygiene, University of Zurich, Zurich, Switzerland; ^3^​ Veterinary Food Safety Laboratory, Cork County Council, Inniscarra, Co. Cork and Department of Microbiology, National University of Ireland, Cork, College Road, Cork, Ireland; ^4^​ Institute for Global Food Security, Queen’s University Belfast, 19 Chlorine Gardens, Belfast BT9 5AG, UK

**Keywords:** comparative genomics, *Yersinia enterocolitica*, Yersinia hibernica, zebrafish embryo

## Abstract

Food-associated outbreaks linked to enteropathogenic *Yersinia enterocolitica* are of concern to public health. Pigs and their meat are recognized risk factors for transmission of *Y. enterocolitica *. This study aimed to describe the comparative genomics of *Y. enterocolitica* along with a number of misclassified *Yersinia* isolates, now constituting the recently described *Yersinia hibernica *. The latter was originally cultured from an environmental sample taken at a pig slaughterhouse. Unique features were identified in the genome of *Y. hibernica,* including a novel integrative conjugative element (ICE), denoted as ICE*_Yh-1_* contained within a 255 kbp region of plasticity. In addition, a zebrafish embryo infection model was adapted and applied to assess the virulence potential among *Yersinia* isolates including *Y. hibernica *.

## Data Summary

This study utilizes sequences previously generated from other studies. *Y. enterocolitica* CFS1932 is deposited in the National Center for Biotechnology Information (NCBI) databases under accession SJZK02000000.

**Impact Statement**

*
Yersinia enterocolitica* is one of the top reported foodborne zoonotic pathogen in the EU. *Yersinia* species can be difficult to identify based on only biochemical tests thus whole-genome sequencing has been increasingly utilized for species identification. *Yersinia hibernica* is a recently described species that is closely related to and commonly misidentified as *Y. enterocolitica *. This study explores the pangenome and comparative genomics of *Y. hibernica* with *Y. enterocolitica *. Genomic analysis reveal the presence of type-III and type-VI secretion systems that are related to those found in pathogenic *Yersinia *. Classical *Yersinia* virulence genes *ail*, *invA*, *hreP*, *myfA*, *vapC*/*vagC* and *ystA* were not identified in *Y. hibernica* however other virulence genes such as *fepA, foxA, ymoA, ystB* and siderophore genes were present. The classical pYV virulence plasmid was not found in any *Y. hibernica* isolates. While *Y. hibernica* has not been associated with human illness, *Y. hibernica* has been isolated from faeces of mammals around the globe. In an *in vivo* zebrafish embryo model, *Y. hibernica* CFS1934 show similar virulence potential to *Y. enterocolitica* 8081. This study is the first in-depth analysis of a novel species of *Yersinia *.

## Introduction

Enteropathogenic *Yersinia enterocolitica* is among the most reported foodborne pathogens in European countries [1]. *Y. enterocolitica* is a rod-shaped Gram-negative, psychrotrophic and facultative anaerobic enteroinvasive pathogen [2, 3] and contamination by this pathogen is of high concern in fattening pigs [4]. This bacterium is one of the main aetiological agents of yersiniosis and most gastrointestinal infections result from consumption of contaminated foods [4, 5]. Thus from a public-health-surveillance standpoint, it is critical to correctly identify *Yersinia* species in suspected yersiniosis cases [6, 7]. However, identification of *Yersinia* is challenging especially in regards to the heterogeneous nature of the *Y. enterocolitica* species [6, 8]. Originally, it was thought that certain biotypes or phylogroups of *Y. enterocolitica* were pathogenic. However, new research studies suggest that all *Y. enterocolitica* are capable of causing disease, even if these strains do not carry the pYV virulence plasmid [4, 5, 9]. Although *Y. enterocolitica* are ubiquitous in nature, these bacteria are often reported from certain types of food products [10] rather than the environment. Palatine tonsils of warm blooded animals, particularly pigs, are identified as one of the important reservoirs of this pathogen [11, 12]. Their occurrence in the environment as well as in the porcine population constitute a threat to public health [13]. Thus, adequate identification and characterization of these diverse bacteria is of practical relevance in understanding its evolution and pathogenicity to humans in the context of the farm-to-fork continuum.

Recently our laboratory conducted a 2-year surveillance programme focused on pigs and their slaughterhouse environment to isolate and characterize porcine-associated *Y. enterocolitica *. Unexpectedly, a novel *Yersinia* species was identified [14]. This novel species, *Y. hibernica* CFS1934, was sequenced to completion and found to be closely related to *Y. kristensenii* and *Y. enterocolitica *. Isolates of *Y. hibernica* have been previously misidentified [15, 16] and have been cultured from animal faeces [17]. While *Y. hibernica* has not been previously associated with human clinical cases, it is currently unknown if the species are non-pathogenic or if previous clinical cases were misidentified as other *Yersinia* species. Indeed pathogenic *Yersinia* have evolved independently within several lineages [18] and *Y. enterocolitica *-like species have been identified in clinical stool samples [19]. In light of this, certain *Y. enterocolitica* (such as biotype 1A) have been found in stools from asymptomatic humans [20, 21], thus identification of *Yersinia* species from stools is not always indicative of pathogenicity, especially if these isolates do not carry the pYV plasmid. However, our laboratory has characterized another novel *Yersinia* species that was recovered from a clinical yersiniosis case where the isolate did not carry the pYV virulence plasmid [22].

These reports support previous studies that the presence of the pYV virulence plasmid alone is not the only defining trait for pathogenicity for yersiniosis, thus a comparative genomic analysis of all *Y. hibernica* isolates to pathogenic *Y. enterocolitica* is warranted. A comparative approach between the newly described *Y. hibernica* and hypervirulent *Y. enterocolitica* will be informative in shedding light on this novel *Y. enterocolitica *-like bacterium.

## Methods

### *Yersinia* strain study collection

*Yersinia* strains (* Y. enterocolitica* subsp. *enterocolitica* 8081, *Y. enterocolitica* subsp. *palearctica* CFS1932, and *Y. hibernica* CFS1934, LC20, IP37048, and CFSAN060539) were selected for genomic comparisons. *Y. enterocolitica* 8081, CFS1932 and CFS0802 along with *Y. hibernica* CFS1934 were used in zebrafish embryo infection studies. *Y. enterocolitica* subsp. *enterocolitica* 8081 (accession AM286415) is a hypervirulent strain isolated from a fatal human septicaemia case [23] and *Y. enterocolitica* subsp. *palearctica* CFS1932 was previously isolated from porcine tonsils and identified as subspecies *palearctica* by the 16S rRNA sequence [24]. *Y. hibernica* CFS1934 was isolated from the lairage floor of a pig slaughterhouse [14].

### Genome analysis

The four *Y. hibernica* isolates and a list of select *Y. enterocolitica* strains (Table S1, available in the online version of this article) were used for a pan-genome comparison. Assembled genomes were annotated by Prokka v1.13.3 [25] and the pan genome was determined by Roary v3.12.0 with a blastp minimum cutoff identity of 90 % to calculate orthologues in the isolates [26, 27]. The UpsetR package was used to visualize intersecting gene sets, representing the core, accessory and pan genome of select *Yersinia* [28]. The ABRicate script (https://git.lumc.nl/bvhhornung/antibiotic-resistance-pipeline/tree/master/tools/abricate) was used to query *Yersinia* sequences for antibiotic resistance with the ResFinder database (updated 28 July 2019) [29] and for virulence genes with the Virulence Finder database (updated 28 July 2019) [30]. Bacterial genomes were also visualized with GView server (https://server.gview.ca/). *Y. hibernica* CFS1934 was set as a reference genome for a blast atlas in which *Y. hibernica* IP37048 [31], *Y. hibernica* LC20 and *Y. hibernica* CFSAN060539 along with *Y. enterocolitica* subsp. *palearctica* CFS1932 and *Y. enterocolitica* subsp. *enterocolitica* 8081 were used to blast against the reference genome. Primers for ICE*Yh*1 are listed in Table S2. Phage regions were determined by https://phaster.ca/ (Table S3) [32]. Gaps in the blast atlas, representing prophage regions and putative genomic islands, were manually inspected to determine gene content. These islands were then coloured and labelled accordingly.

### *In vivo* zebrafish embryo infection studies

The *wik* lines of zebrafish (*Danio rerio*) were used in this study. Adult zebrafish were kept at a 14/10 h light/dark cycle at a pH of 7.5 and 27 °C. Adult zebrafish were set up pairwise in individual breeding tanks for natural spawning and egg collection. Embryos were raised in petri dishes containing E3 medium (5 mM NaCl, 0.17 mM KCl, 0.33 mM CaCl_2_, 0.33 mM MgSO_4_) supplemented with 0.3 µg ml^−1^ methylene blue at 28 °C. From 24 h post-fertilization (h p.f.), 0.003 % 1-phenyl-2-thiourea (PTU) was added to prevent melanin synthesis. Staging of embryos was performed according to Kimmel *et al.* [33]. Bacterial inoculum preparation was performed following the procedure described by Eshwar *et al.* [34].

### Zebrafish embryos infection by microinjection

Injections of bacterial were conducted using borosilicate glass microcapillary injection needles (Science Products GmbH, Hofheim, Germany) and a PV830 Pneumatic PicoPump (World Precision Instruments, Sarasota, FL, USA). Prior to injection, embryos at 2 days post-fertilization (days p.f.) were manually dechorionated and anesthetized with 200 mg l^−1^ buffered tricaine (Sigma-Aldrich, Buchs, Switzerland). Afterwards, the embryos were aligned on an agar plate and injected with ~500 colony forming units (c.f.u.) in a 1–2 nl volume of a bacterial suspension in Dulbecco's phosphate-buffered saline directly into the blood circulation (caudal vein). The number of c.f.u. injected at 0 h post-infection (h p.i.) was determined by manually disintegrating five euthanized embryos individually after microinjection of bacteria (0 h p.i.) then plated, which resulted in an accurate determination of the number of c.f.u. actually injected.

Following injection, the infected embryos were allowed to recover in a petri dish with fresh E3 medium for 15 min. For survival assays, embryos were transferred into single wells of a 24-well microplate containing 1 ml of E3 medium per well, incubated at 28 °C and observed for survival under a stereomicroscope twice a day. Virulence was assessed by determination of the survival rate (30 embryos: 10 embryos per bacterial strain, in three independent experiments) over 72 h p.i. (=3 days post-infection, days p.i.). The number of dead embryos was determined visually based on the absence of heartbeat.

### Ethics statement

Zebrafish husbandry was conducted with approval (Licence Number 150) from the Veterinary Office, Public Health Department, Canton of Zurich (Switzerland) allowing experiments with zebrafish embryos and larvae <5 days p.f. Zebrafish embryos at this age have not reached the free feeding stage and pain sensitivity is not developed at 4–7 days p.f. in accordance with animal ethics. Zebrafish larvae mortality was assessed at selected time points visually based on the lack of a heartbeat. Experiments carried out until 72 h p.i. and post-experiments embryos were euthanized by incubation in 4 g l^−1^ buffered tricaine solution for at least 10 min and/or until cessation of opercular movement.

### Statistical analysis

Kaplan Meier survival analysis and statistics [Log-rank (Mantel–Cox) test] for zebrafish embryo infection experiments were performed with GraphPad Prism 7 (GraphPad Software, CA, USA). One way ANOVA with post-hoc Tukey HSD tests were used to assess the statistical significance.

## Results and discussion

### Core *Y. hibernica* genome and virulence genes

**Fig. 1.**
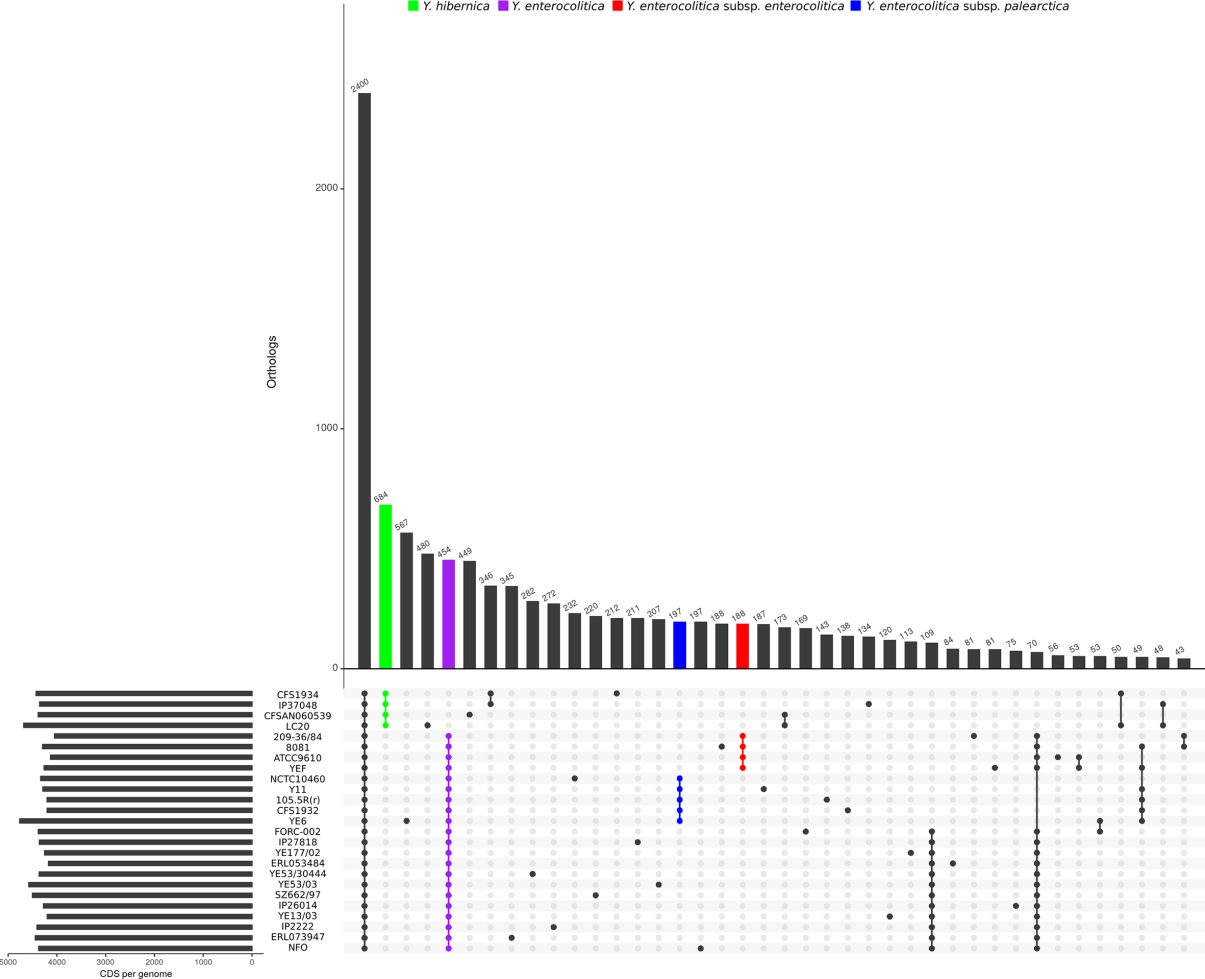
Representation of the core and pan genome of *Y. hibernica* (green) in comparison to *Y. enterocolitica* (purple) as determined by Roary with a blastp 90 % identity cutoff for orthologous genes. *Y. enterocolitica* subsp. *enterocolitica* (red) and *Y. enterocolitica* subsp. *palearctica* (blue) are also represented in the core genome.

As the identification of a novel *Yersinia* species resulted from an earlier study, a genomic comparison between *Y. hibernica* and *Y. enterocolitica* was warranted. Three other *Y. hibernica* were identified in public reference databases including strains LC20 (NZ_CP007448), CFSAN060539 (NZ_NHOF00000000) and IP37048 (CABHXI000000000). Within the McNally seven-gene MLST scheme [35], *Y. hibernica* IP37048 and CFS1934 are identified as ST334 whilst LC20 and CFSAN060539 are ST217. An UpsetR plot (Fig. 1) was constructed to visualize CDS intersections representing the core and pan genome of *Y. hibernica* and a select number of *Y. enterocolitica* based on a blastp cutoff of 90 % identity. A total of 2400 core genes were identified between the two species with *Y. hibernica* encoding 684 unique core genes as determined by Roary (green bar, Fig. 1). In total, 454 core genes were identified in *Y. enterocolitica sensu stricto* (purple bar, Fig. 1) with 197 core genes specific to *Y. enterocolitica* subsp. *palearctica* (blue bar, Fig. 1) and 188 core genes specific to *Y. enterocolitica* subsp. *enterocolitica* (red bar, Fig. 1).

**Fig. 2.**
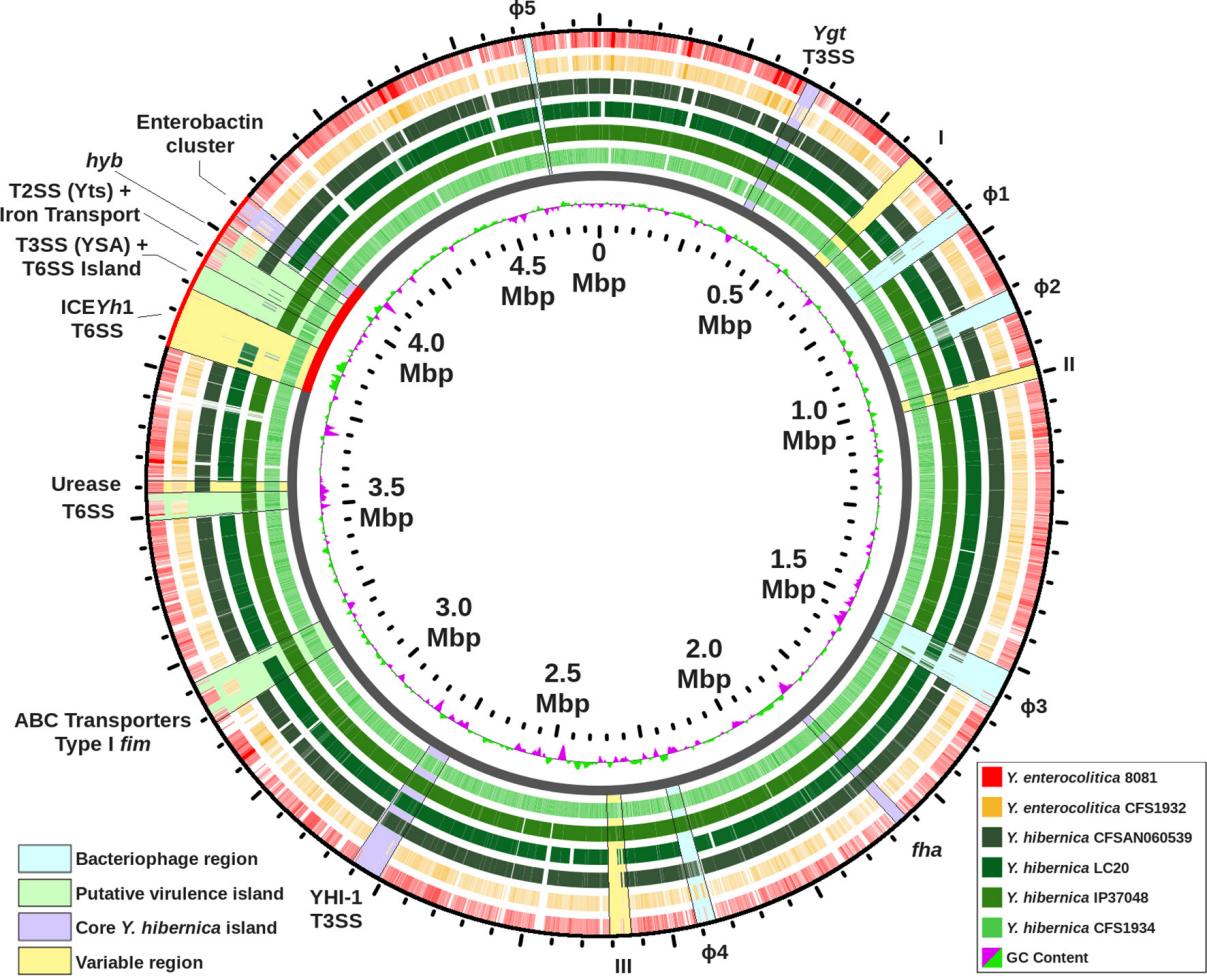
blast atlas with *Y. hibernica* CFS1934 set as the reference genome (inner ring). All *Y. hibernica* strains represented in the figure are in various shades of green. The hypervirulent *Y. enterocolitica* subsp. *enterocolitica* 8081 is shaded in red and the environmental *Y. enterocolitica* subsp. *palearctica* CFS1932 is shaded in yellow. The plasticity zone is indicated by the red backbone.

A blast atlas of *Y. hibernica* CFS1934, IP37048, LC20 and CFSAN060539 was constructed in comparison to *Y. enterocolitica* CFS1932 and 8081 (Fig. 2). *Y. hibernica* LC20 and CFSAN060539 (also known as strain FCF 604) were originally misidentified as *Y. enterocolitica* [15] and *Y. kristensenii* respectively [17], but on the basis of nucleotide identity and 16S rRNA gene identity, these have been reclassified as *Y. hibernica* [14].

The blast atlas revealed regions that are unique to *Y. hibernica* (denoted in Fig. 2, as purple regions). Using the locus tag numbers from *Y. hibernica* CFS1934 as the reference, the Ygt island (D5F51_RS01695–D5F51_RS01850) encodes a type-III secretion system (T3SS) similar to that described in an Old-World biotype 3 *Y. enterocolitica* and in other *Yersinia* lineages [18, 36]. The Ygt T3SS is believed to be ancestral within members of *Yersinia* [37] and was found to have been lost from acute pathogenic members of *Y. enterocolitica* and *Y. pestis* [18]. Interestingly, a Ygt T3SS mutant constructed in *Y. enterocolitica* showed no differences in virulence when tested in a *Galleria mellonella* infection model [5].

**Fig. 3.**
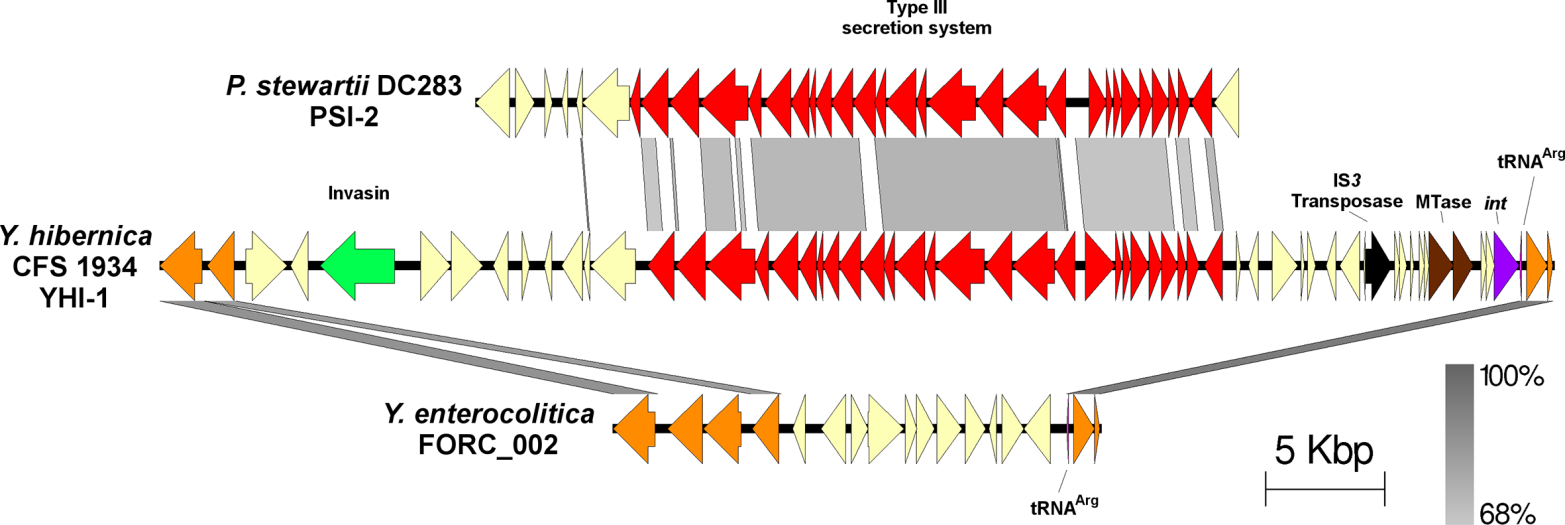
YHI-1 encodes a T3SS module (red) that is distantly related to PSI-2 (* Pantoea* secretion island 2) in the plant pathogen *P. stewartii.* YHI-1 encodes an invasin (green).

A novel island, denoted as YHI-1 (* Y. hibernica* island 1, D5F51_RS13065–D5F51_RS13335), encoding a separate T3SS, was located in the chromosome of all *Y. hibernica* (Fig. 2). YHI-1 appears to be horizontally acquired in the common ancestor of *Y. hibernica* as the corresponding region in related *Y. enterocolitica* is devoid of this sequence (blastn, Fig. 3). An integrase gene was found to be located adjacent to a tRNA^Arg^, however no *attL* nor *attR* repeats were identified. The YHI-1 T3SS is similar to the *Pantoea* secretion island 2 (PSI-2) found in *Pantoea ananatis* and *Pantoea stewartii* subsp. *stewartii* [38] and an undescribed type-III secretion system in *Cedecea neteri* [39] (Fig. 3). *C. neteri* is a rare isolate from immunocompromised human clinical cases [40] while *Pantoea* are plant pathogens that cause rare opportunistic infections in humans [41]. The PSI-2 of *P. stewartii* subsp* . stewartii* is part of the Inv-Mxi-Spa T3SS family, which are generally associated with animal pathogens [38, 42]. PSI-2 is required for persistence of *P. stewartii* subsp* . stewartii* in flea beetle vectors.

An island (*fha*, D5F51_RS08570–D5F51_RS08620) encoding a type-I fimbrial operon and filamentous haemagglutinin was present in all *Y. hibernica *. A blastn analysis revealed the *fha* island was only present in one other strain in NCBI GenBank, *Y. frederiksenii* FDAARGOS_418. The island was likely acquired via horizontal gene transfer as it possesses a 38.1 % GC content, however no transposons nor insertion sequences were identified flanking the island.

Of note, the occurrence of an ampicillin resistance-encoding gene was reported, according to the literature, in isolates of non-clinical origin [43], a feature that was supported by the findings reported herein. *Y. enterocolitica* 8081 encodes two β-lactamases located at different positions on the bacterial chromosome. These are denoted as *blaA* (YE2019) and *blaB* (YE2440) the former of which expresses resistance to penicillins and cephalosporins [44]. *Y. hibernica* CFS1934 does not encode *blaB* but does encode a novel class A β-lactamase (D5F51_RS08775) that shares 85.03 % amino acid identity with the *blaA* gene product in *Y. enterocolitica* 8081. blastn analysis shows that this *bla* gene is present in IP37048 but truncated in CFSAN060539 and LC20 (Fig. S1). Similar to other described *Yersinia* BlaA, the *Y. hibernica* CFS1934 *bla* gene has a twin-arginine translocation (Tat) motif for Tat-dependent secretion [45]. Susceptibility of *Y. hibernica* CFS1934 to a panel of antimicrobial compounds performed using disc diffusion revealed it is resistant to ampicillin (data not shown).

In previous studies, *Y. hibernica* LC20 and CFS1934 were reported in its inability to utilize sucrose [14, 15]. Analysis of the sucrose porin operon in closely related *Y. canariae* and *Y. enterocolitica* reveal a deletion of the transporter operon in *Y. hibernica* (Fig. S2). blast analysis of *Y. hibernica* isolates shows that all sequenced isolates share this deletion. Indeed, sucrose-negative *Y. enterocolitica-*like isolates have been reported from environmental sources [46] but without sequencing [8] and since the 16S rRNA gene for *Y. enterocolitica* and *Y. hibernica* is very similar [14], it is nearly impossible to determine if previous reports of sucrose-negative *Y. enterocolitica* were potentially misidentified.

In addition to regions of differences observed in the blast atlas, all *Y. hibernica* strains were queried for known *Yersinia* virulence factors [17, 47] via the Virulence Factor Database [24]. Classical *Yersinia* virulence genes *ail*, *invA*, *ystA*, *hreP*, *myfA* and *vapC*/*vagC* were not identified in *Y. hibernica* [48]. In contrast, *Y. hibernica* had homologues of classical virulence genes *ymoA* (D5F51_RS13625), heat-stable enterotoxin-encoding *ystB* (D5F51_RS06925) [49], *fepA* (D5F51_RS19110) and *foxA* (D5F51_RS01865) of which the latter two are involved in iron transport and uptake in *Y. enterocolitica* [50]. While the *fepA* gene in *Y. enterocolitica* 8081 was reported to be a pseudogene [19], the *fepA* gene in *Y. hibernica* is intact. Indeed, the entire enterochelin *fep* locus was present in *Y. hibernica* (D5F51_RS19070–D5F51_RS19110) [51]. Additionally the enterobactin transporter *entS* gene (D5F51_RS19075) in the *fep* locus in *Y. hibernica* is not similar to the *fes* gene in *Y. enterocolitica* 8081, but it is related to those identified in *Y. canariae, Y. kristensenii* and *Y. frederiksenii *. The *yfe* locus (D5F51_RS07700–D5F51_RS07715) encoding for an ABC iron/manganese transporter system and the *feo* iron transporter system (D5F51_RS20970–D5F51_RS20985) were also present in all *Y. hibernica.* The *yfe* locus is important for full virulence in *Y. pestis* [52, 53] while the *feo* locus was shown to be important in bubonic plague progression in *Y. pestis* [54]. These siderophore uptake and utilization genes are critical for survival in iron-poor environments such as the gut lumen [51]. The product of the *Yersinia* modulator gene, *ymoA*, thermoregulates the expression of virulence genes such as invasin [55, 56] and type-III secretion systems in *Y. pestis* [57]. The flagellar export apparatus along with chemotaxis genes were identified in all *Y. hibernica* (D5F51_RS10660–D5F51_RS10870) [9, 58]. The YplA phospholipase is transported by the flagellar type-III secretion system and a similar phospholipase (D5F51_RS16365) was identified in *Y. hibernica* [58, 59]. The phospholipase is linked to bacterial colonization by *Y. enterocolitica* of mice Peyer’s patch and modulated pro-inflammatory responses [59].

A homologue of a novel intimin family protein Ifp in *Y. pseudotuberculosis* that is capable of binding human HEp-2 epithelial cells [60] was identified in *Y. hibernica* (D5F51_RS13630). An invasin-like gene was detected (D5F51_RS13075) with no close homologues being identified by NCBI blast. An omptin-encoding gene (D5F51_RS07435) is present on the chromosome of *Y. hibernica* and shares 63.07 % amino acid identity with the plasmid-encoded Pla plasminogen activator from *Y. pestis* but is unlikely to have similar biological functions [61]. Another hydrogenase cluster (D5F51_RS11850–D5F51_RS11930), which encodes the Hyd-2 and Hyd-4 complexes (*hyf* locus) were also present in all *Y. hibernica* strains [17, 19]. A pseudogene encoding the novel insecticidal enterotoxin YacT was identified (D5F51_RS09845) but it is interrupted by an IS*1* family transposase in *Y. hibernica* CFS1934 and IP37048, however it is intact in LC20 and CFSAN060539 [62]. Additionally, a putative type-III effector (D5F51_RS05270) similar to the *Salmonella* NleB was identified in *Y. hibernica* [63], however it is unknown if it plays a similar function.

### Variable regions within the *Y. hibernica* genome

Other regions of differences were also identified and found to be present only in certain strains of *Y. hibernica* (denoted by the yellow coloured regions, highlighted in Fig. 2). Region I (D5F51_RS02760–D5F51_RS02915) contains an island that encodes a putative orphan DNA cytosine methyltransferase (D5F51_RS02805) found only in *Y. hibernica* CFS1934 and closely related *Y. hibernica* IP37048. Region II (D5F51_RS04705–D5F51_RS04800) encodes a filamentous haemagglutinin (D5F51_RS04775) with similarities to those previously described and encoded by *Y. massiliensis* and was only found in *Y. hibernica* CFS1934 and IP37048. Region III (D5F51_RS11020–D5F51_RS11145) shares a similar *int* (D5F51_RS11020) with the *Y. enterocolitica* high-pathogenicity island (HPI) and is present in *Y. hibernica* CFS1934 and IP37048. Region III also encodes a Bcr/CflA efflux MFS transporter (D5F51_RS11055) but does not have much similarity with the *Y. enterocolitica* HPI. The region-III island has similarities with those identified in *Serratia quinivorans* and *S. ficaria* but not much else is known about the island (Fig. S3).

A urea utilization operon (D5F51_RS16710–D5F51_RS16675) was identified in all *Yersinia* except for *Y. hibernica* LC20, which was previously reported to be deficient in urea utilization [15]. Adjacent to the urea operon in *Y. hibernica* CFS1934 and IP37048 is a type-VI secretion system island (D5F51_RS16560–D5F51_RS16420) that is related to those found in *Y. intermedia* and *Y. rohdei* [64]. Various ABC transporters and a type-I fimbrial operon (D5F51_RS15015–D5F51_RS15045) were not present in *Y. hibernica* CFSAN060539 but were present in other *Y. hibernica *.

The phaster tool [26] was used to identify phage regions within *Y. hibernica* CFS1934 and visual inspection of the genome was used to determine the *attL* and *attR* boundaries of the bacteriophages (Fig. 2, light blue). No putative virulence cargo were identified in the phage genomes. Plasmid pCFS1934 (accession NZ_CP032488) was queried by the PLSDB plasmid database [65] to identify any closely related plasmids by Mash distance using default parameters and none were identified (data not shown). A blastn search of pCFS1934 against all *Y. hibernica* sequences indicated *Y. hibernica* IP37048 likely has a related plasmid (data not shown).

### *Y. hibernica* plasticity zone

**Fig. 4.**
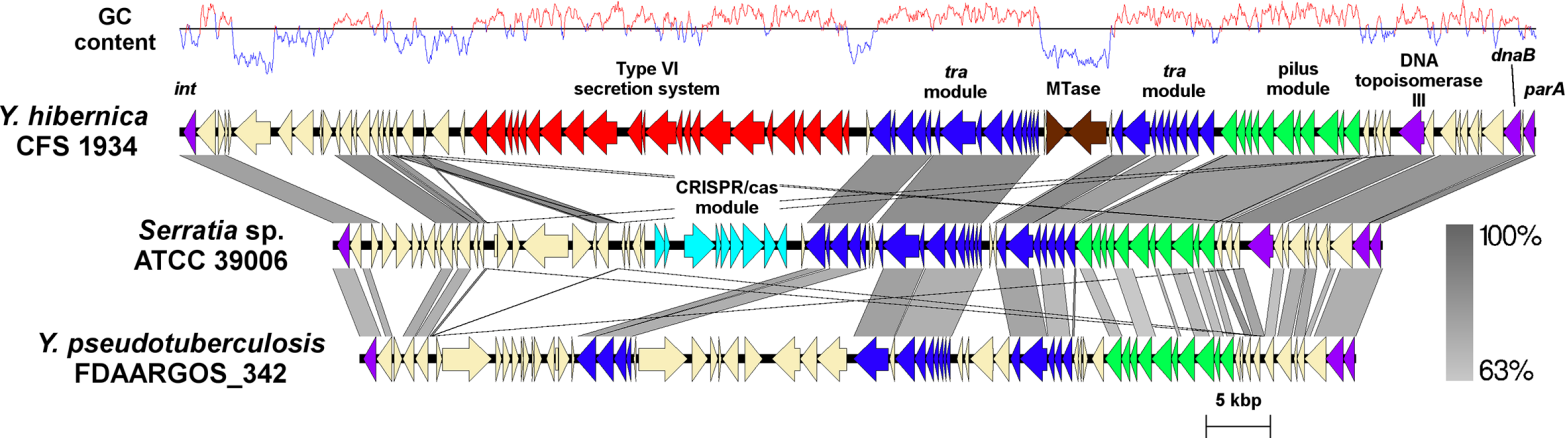
ICE*Yh*1 compared to related ICEs from other species with shared conjugative transfer modules (blue), ICE replication (purple) and pilus (green). Notably, ICE*Yh*1 carries a T6SS module (red) and DNA methylase (brown) within its cargo.

Similar to *Y. enterocolitica *, a plasticity zone was identified in *Y. hibernica* (Fig. 2, red backbone). A 106.2 kbp integrative conjugative element (ICE, denoted as ICE*Yh*1) was found to be integrated between nucleotide positions 3 787 208–3 893 449 in *Y. hibernica* CFS1934 alone and within a 255 kbp plasticity zone. Similar to other known ICEs, the attachment encoding locus *attB* for ICE*Yh*1 is located within the tRNA^Phe^ gene, resulting in a 49 bp duplication of the 3′-end of the tRNA^Phe^ gene [66]. The *int* gene (D5F51_RS17855) encodes a putative tyrosine recombinase, much like other ICE integrases. Primers flanking the putative *attL* and *attR* of this ICE (Table S2) were used to determine whether or not the putative ICE could excise from the genome. The *attB* on the chromosome and *attP* on the ICE were detected after PCR amplification, suggesting the ICE can be mobilized (data not shown).

blastn analysis of ICE*Yh*1 revealed distantly related ICEs in several *Y. pseudotuberculosis* and *Serratia* species ATCC 39006 [67] (Fig. 4). These related ICE also integrate into the 3′-end of the tRNA^Phe^ gene, likely due to the conserved integrase (Fig. 4). Despite carrying similar modules for integration, conjugation and transfer, ICE*Yh*1 possesses distinct cargo modules when compared to the ICE in *Y. pseudotuberculosis* and *Serratia* species ATCC 39006.

A putative type-II DNA methyltransferase (D5F51_RS18160) and a putative type IIG restriction enzyme/DNA methyltransferase (D5F51_RS18165) are embedded within the *tra* module in ICE*Yh*1 [68]. A type-VI secretion system (T6SS) cluster (D5F51_RS17960–D5F51_RS18070) is also present in ICE*Yh*1. A blastn search of the ICE*Yh*1 T6SS shows similarities to one found in *Y. hibernica* LC20 (Fig. 2) and some similarities to those identified in *Y. pseudotuberculosis* (data not shown). As the Hcp effector is a hallmark of T6SS [69], the ICE*Yh*1 Hcp-encoding gene (D5F51_RS18045) was queried in NCBI by blastp. The ICE*Yh*1 Hcp is identical to the *hcp1* and *hcp2* gene products found in *Y. pestis* CO92, which are encoded by T6SS cluster B and cluster C, respectively [70]. Using an *in vivo* mouse model of infection, Andersson *et al.* [70] demonstrated that deletion mutants of Hcp1 and the cluster B T6SS resulted in significant attenuation of *Y. pestis* CO92.

Interestingly, the *ysa* type-III secretion island (D5F51_RS18510–D5F51_RS18665), the Yts general secretion pathway (type-II secretion system) operon (D5F51_RS18685–D5F51_RS18745), and an additional iron-transport cluster (D5F51_RS18815–D5F51_RS18830) that resembles the *fit* locus from *Y. pestis* were present in the plasticity zone [23, 71] in *Y. hibernica* CFS1934, IP37048 and *Y. enterocolitica* 8081 [18]. The *fit* locus has been detected in *Y. pestis* and *Y. pseudotuberculosis* but has not been tested for virulence [72]. However, the yersiniabactin loci (YE2612–YE2622 in *Y. enterocolitica* 8081) was not identified in *Y. hibernica *. The *hyb* locus (D5F51_RS18940–D5F51_RS18995), which encodes for the hydrogenase complex loci, linked to be essential for gut colonization [23], was present in the plasticity zone of all *Y. hibernic *a. Despite the lack of *ail* or *inv* genes being found in *Y. hibernica *, the presence of siderophores, enterotoxins, invasins, hydrogenase utilisation clusters, iron transporters and type-II/type-III secretion systems suggest that this species may be capable of surviving in iron-poor gut lumens. The potential ability to regulate inflammatory responses by phospholipases and to regulate virulence genes with YmoA suggest an ability to navigate mammalian gut lumens and indeed *Y. hibernica* strains have been isolated from rodent faeces [14, 16]. Nonetheless at this time, it is unknown if *Y. hibernica* can be considered as human pathogens.

### Pathogenicity of *Y. hibernica* and *Y. enterocolitica* explored using a zebrafish embryo infection model

A zebrafish embryo model of infection was adapted to assess the potential pathogenicity of *Y. hibernica* CFS1934 and selected *Y. enterocolitica* of varying virulence genotypes (Fig. S4). Survival experiments were carried out on zebrafish infected with *Y. hibernica* CFS1934, *Y. enterocolitica* 8081, *Y. enterocolitica* CFS0802 and *Y. enterocolitica* 1932. At 3 days p.i., a survival rate of 50 % was observed in embryos injected with *Y. hibernica* CFS1934 as well as *Y. enterocolitica* 8081, 60 % with *Y. enterocolitica* CFS0802 and 40 % with *Y. enterocolitica* CFS1932. Survival rates in injections using DPBS (as well as uninjected controls) remained unaltered. Based on these observations, infection with *Y. hibernica* CFS1934 resulted in a similar reduction in survival rate of the embryos in comparison to the hypervirulent *Y. enterocolitica* 8081. The latter results suggested that *Y. hibernica* CFS1934 exhibited a virulence potential, similar to the one of the hypervirulent *Y. enterocolitica* strain 8081. Previous infection studies in *Galleria mellonella* show that *Y. enterocolitica* pathogenicity is enhanced at lower temperatures (25 °C) when compared to 37 °C [5]. As Alenizi *et al.* previously suggested [5], all *Y. enterocolitica* are pathogenic and in a zebrafish embryo infection model, *Y. hibernica* shows a similar virulence potential to the *Y. enterocolitica* strains tested.

## Conclusions

As *Y. hibernica* has only been recently identified and characterized, a comparative genomics study detailing the virulence potential of this organism with other, better characterized *Yersinia* will provide an understanding of this species. The comparative genomics detailed in this work show that certain classical *Yersinia* virulence orthologous genes are present in this species. The presence of iron-transport clusters and other siderophores suggest an ability of *Y. hibernica* to survive in iron-deficient environments such as the mammalian gut niche. Additionally, the initial cold enrichment for the *Y. hibernica* type strain CFS1934 and subsequent studies at 25 and 37 °C indicate this *Yersinia* species, similar to other described *Yersinia *, is capable of growth in a broad range of temperatures that is relevant in an environmental and mammalian warm-body context. *Y. hibernica* have only been isolated from the environment with LC20 from *Rattus norvegicus* faeces and CFSAN060539 from *Hydrochoerus hydrochaeris* faeces, thus it is tempting to suggest a link between *Y. hibernica* and mammalian environments. *Y. hibernica* isolates have been misidentified as *Y. enterocolitica* and *Y. kristensenii* in previous studies, highlighting the difficulty in identifying *Yersinia* species through biochemical methods. A literature search reveals scattered reports of *Y. enterocolitica *-like isolates from environmental to human sources that do not utilize sucrose, however without sequencing data, it is impossible to determine what *Yersinia* species was isolated. Despite this, the association of *Y. hibernica* with mammalian hosts in proximity with humans and the presence of potential virulence factors warrants a closer monitoring of this species, to protect public health.

## Supplementary Data

Supplementary material 1

Supplementary material 2
