# Supplementary material for: Comparative genomic insights into Yersinia hibernica – a commonly misidentified Yersinia enterocolitica-like organism
Source: Microb Genom. 2020 Jul 23;6(9):mgen000411. doi: 10.1099/mgen.0.000411 (PMC7643974; doi:10.1099/mgen.0.000411)
Supplement: Supplementary material 1 [file mgen-6-411-s001.pdf]

Figure S1

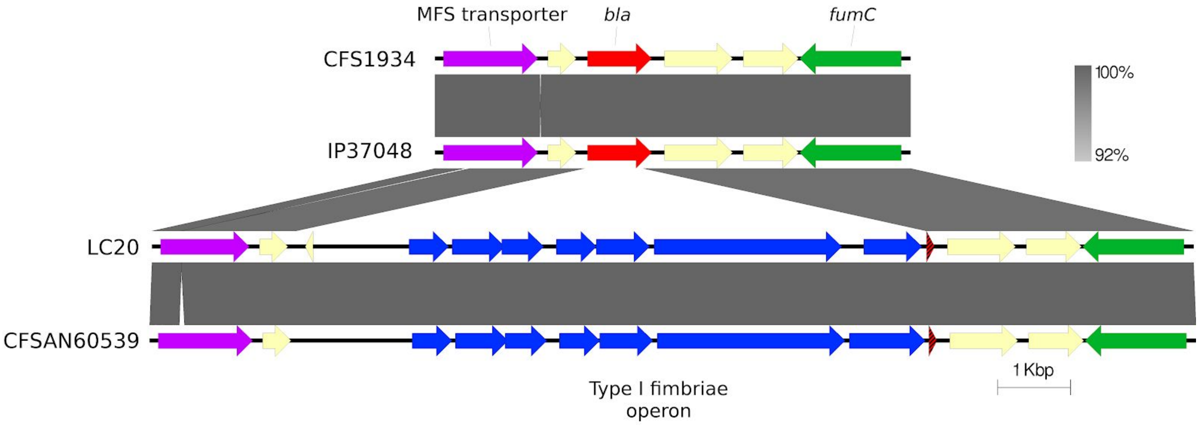

Insertion of a type I fimbriae operon in *Y. hibernica* LC20 and CFSAN60539 interrupted a novel *bla* gene (red hatched) that is intact in other *Y. hibernica* strains (red).

Figure S2

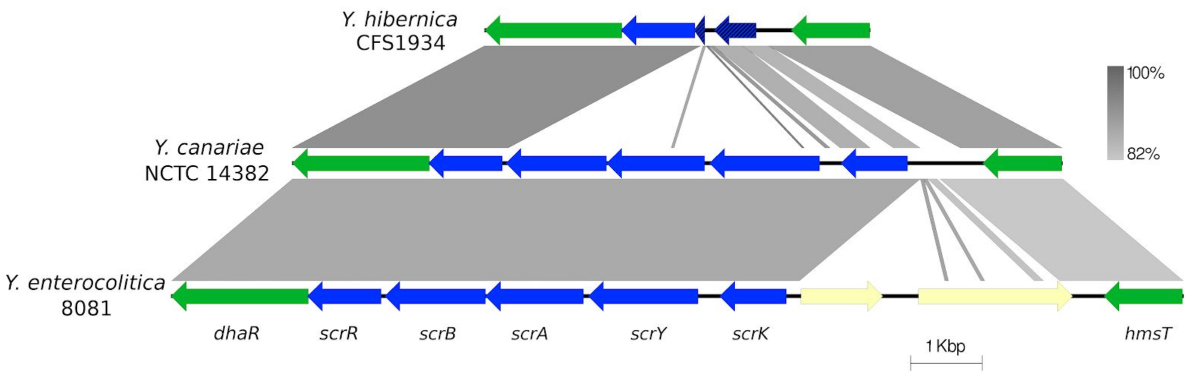

The sucrose transporter operon is deleted in all *Y. hibernica* (*scrB, scrA, scrY,* and *scrK*). Closely related *Y. canariae* shows the presence of the operon. Synteny of the region is conserved when compared to *Y. enterocolitica*.

Figure S3

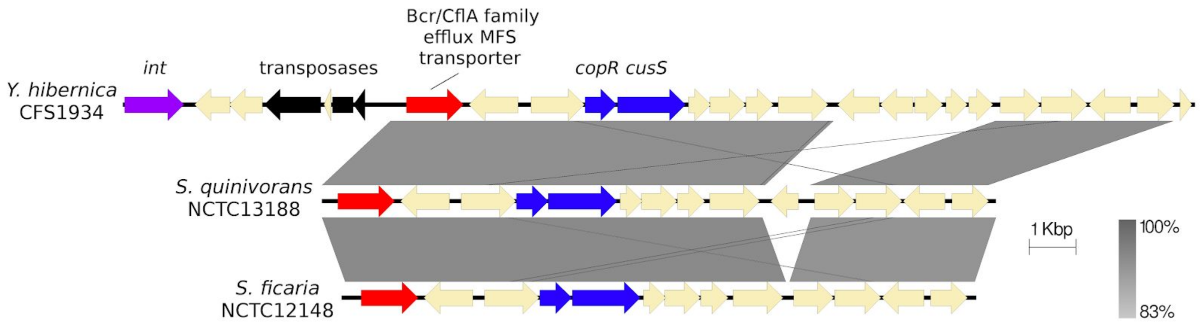

Region III is present in *Y. hibernica* CFS1934 and IP37048 and encodes a Bcr/CflA efflux MFS transporter (D5F51_RS11055). This region is present in other members of *Yersiniaceae*, such as *Serratia* species.

Figure S4

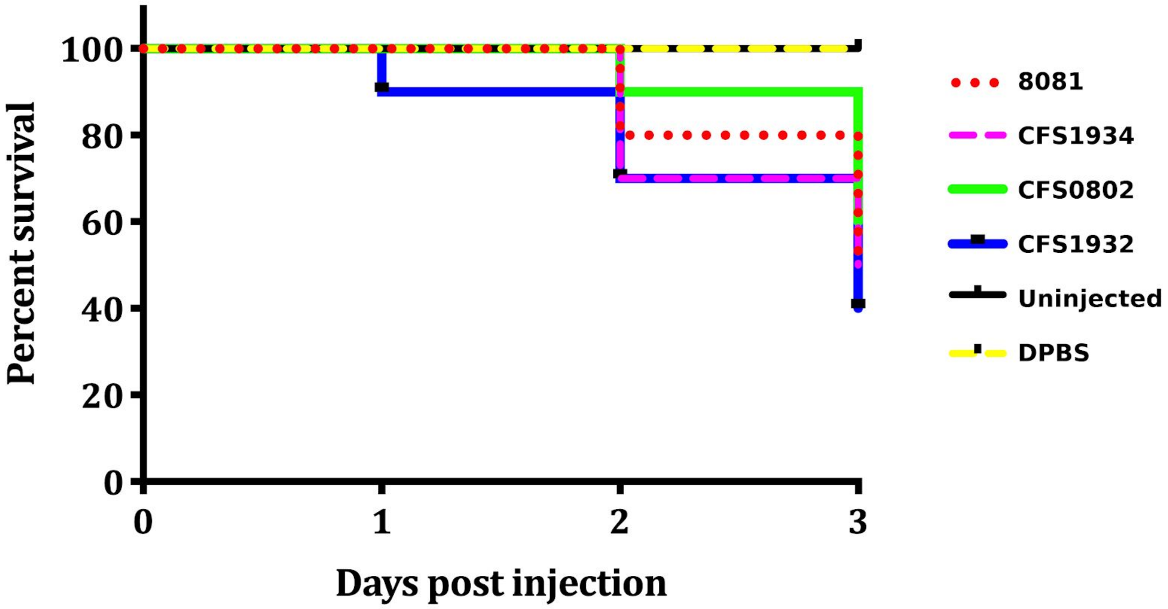

Survival data of zebrafish larvae infected with *Y. hibernica* CFS1934, *Y. enterocolitica* 8081, *Y. enterocolitica* CFS0802, and *Y. enterocolitica* CFS1932 and controls over 72 hpi. DPBS, Dulbecco's phosphate-buffered saline.
